# Family-Specific Degenerate Primer Design: A Tool to Design Consensus Degenerated Oligonucleotides

**DOI:** 10.1155/2013/383646

**Published:** 2013-02-21

**Authors:** Javier Alonso Iserte, Betina Ines Stephan, Sandra Elizabeth Goñi, Cristina Silvia Borio, Pablo Daniel Ghiringhelli, Mario Enrique Lozano

**Affiliations:** ^1^LIGBCM-Área Virosis Emergentes y Zoonóticas, Universidad Nacional de Quilmes, B1876BXD Buenos Aires, Argentina; ^2^LIGBCM-Área Virosis de Insectos, Universidad Nacional de Quilmes, B1876BXD Buenos Aires, Argentina

## Abstract

Designing degenerate PCR primers for templates of unknown nucleotide sequence may be a very difficult task. In this paper, we present a new method to design degenerate primers, implemented in family-specific degenerate primer design (FAS-DPD) computer software, for which the starting point is a multiple alignment of related amino acids or nucleotide sequences. To assess their efficiency, four different genome collections were used, covering a wide range of genomic lengths: *Arenavirus* (10 × 10^4^ nucleotides), *Baculovirus* (0.9 × 10^5^ to 1.8 × 10^5^ bp), *Lactobacillus* sp. (1 × 10^6^ to 2 × 10^6^ bp), and *Pseudomonas* sp. (4 × 10^6^ to 7 × 10^6^ bp). In each case, FAS-DPD designed primers were tested computationally to measure specificity. Designed primers for *Arenavirus* and *Baculovirus* were tested experimentally. The method presented here is useful for designing degenerate primers on collections of related protein sequences, allowing detection of new family members.

## 1. Introduction

The polymerase chain reaction (PCR), one of the most important analytical tools of molecular biology, allows a highly sensitive detection and specific genotyping of environmental samples, specially important in the metagenomic era [1]. A large list of genome typing applications includes arbitrarily primed PCR [2] (AP-PCR), random amplified primed DNAs [3] (RAPDs), PCR restriction fragment length polymorphism [4] (PCR-RFLP), and direct amplification of length polymorphism [5] (DALP). All of these techniques require a high quality and purity of the specific target template, because any available DNA could be substrate for the amplification step. In view of this, genotyping procedures of large genomes or complex samples are more reliable if they are based on DNA amplification using specific oligonucleotides. Therefore, primer design is crucial for efficient and successful amplification.

Several primer design programs are available (e.g., OLIGO [6], OSP [7, 8], Primer Master [9], PRIDE [10], Primer3 [11], among others). Regardless of each computational working strategy, all of these use a set of common criteria (e.g., *G*/*C* content, melting temperature, etc.) to evaluate the quality of primer candidates in a specific target region selected by the user. Alternative programs are aimed at more specific purposes, such as selection of primers that bind to conserved genomic regions based on multiple sequence alignments [12, 13], primer design for selective amplification of protein-coding regions [14], oligonucleotide design for site-directed mutagenesis [15], and primer design for hybridization [16]. Usually, the design of truly specific primers requires the information of the complete nucleotide sequence. This is the starting point for most of the programs described in the literature. However, the need of designing specific primers is not always accompanied by the complete knowledge of the target genome sequence.

A primer, or more generally any DNA sequence, is called specific if it represents a unique sequence and is called degenerate if it represents a collection of unique sequences. For example, the amino acid sequence “YHP” could be coded by “TATCATCCC,” “TACCATCCA,” or “TACCACCCG,” among others; all of these are unique sequences that can be summarized in a “degenerate” nucleotide sequence “TAYCARCCN,” using IUPAC code. Operatively, the use of a degenerate primer implies the use of a population of specific primers that cover all the possible combinations of nucleotide sequences coding for a given protein sequence. Also, primers including modified bases can be used. Some modified bases can match different bases.

Although the increase in degeneracy rises the chance of unspecific annealing of the designed primers, it also increases the probability of finding unknown divergent variants of a sequence family. This dual behavior must be taken into account during the design. Algorithmic search of primers that include degenerated positions is usually defined as the degenerate primer design (DPD) problem. In recent years, several methods were developed to solve DPD problem. Each one has a specific scope or is designed to solve a variant of the problem, but all of them aim to minimize the number of degenerations of the resulting primers.

The DPD problem was expressed in different ways by many researchers. Linhart and Shamir [17] presented the maximum coverage DPD problem (MC-DPD), with the goal of finding a primer that covers the maximum number of input sequences. The selection of primers is constrained by limiting the maximum degeneracy. They also stated the minimum degeneracy DPD problem (MD-DPD), in which the objective is finding a primer with the minimum degeneracy that covers all the input sequences. To solve MC-DPD they have developed the HYDEN program [18]. Wei et al. [19] developed the DePiCt program that uses hierarchical clustering of protein blocks to design the primers. Rose et al. [20] developed a method for hybrid degenerate-nondegenerate primers, where the 3′ region is degenerated and its 5′ region is a consensus clamp. It was implemented in CODEHOP [21] and iCODEHOP [22] programs and was used to search new members of protein families and for identification and characterization of viral genomes. Balla and Rajasekaran [23] described a method for a variant of MD-DPD that tolerates mismatch errors, implemented in the minDPS program. The programs PT-MIPS and PAMPS address mainly the problem of multiple degenerate primer design. The aim of these programs is finding the minimum number of degenerate primers that cover all the input sequences, taking into account that none of them may be more degenerated than an input value.

In this study a new method for solving the DPD problem is proposed, in which the focus is shifted away from the global minimum degenerated primer in favor of maximizing a score value which contains degeneracy but weighted by its proximity to the 3′ end of the primer. This minimizes the degeneracy at that end while allowing more freedom in the remaining positions. Hereby, the best scoring primers may not be the less degenerated, but take into account a biological restraint that is not so heavily considered in other methods. The 3′ end is the essential anchoring site because it is where the polymerase initiates its activity. From a strategic point of view, a decision must be made whether or not to allow degeneracy at this end. The presence of degeneracy at the 3′ end probably assures a greater diversity of sequences to be detected. However, at the same time, it diminishes the proportion of primer specific for a given sequence. Therefore, we decided to be very strict in the search of conserved regions and minimize the amount of degeneracy incorporated at this end. If the input set of sequences is sufficiently large, it is highly probable that a region identified as conserved among all known sequences will likewise be conserved in any new member of the family. 

## 2. Scoring and Primer Search Strategy

**Figure 1:**
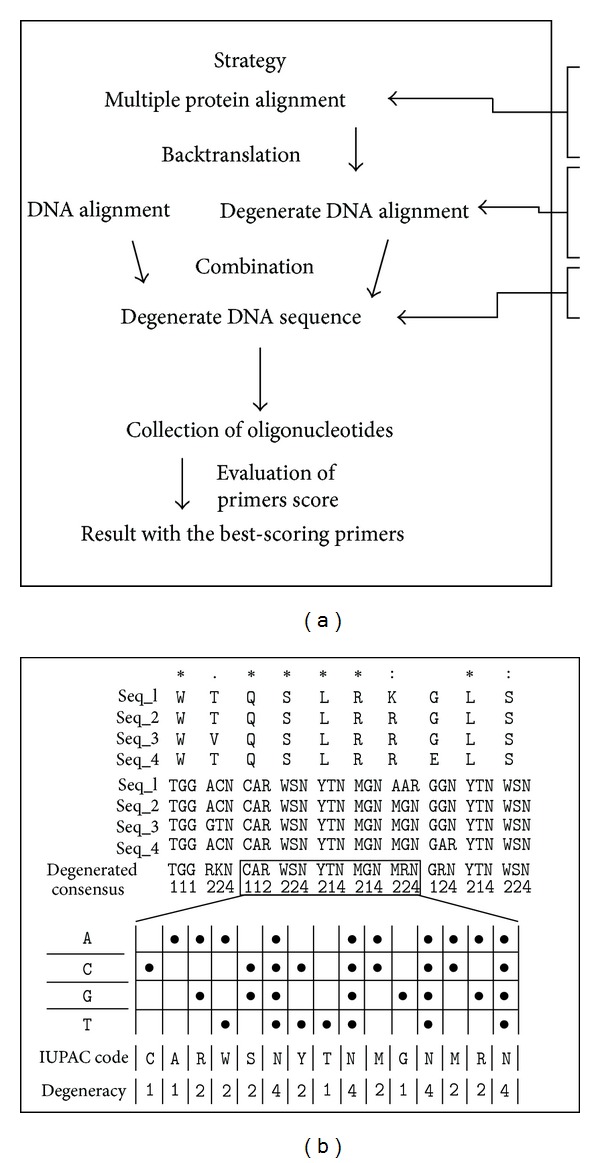
Minimum degenerated sequence generation. (a) Diagram of the general strategy used. (b) Sample protein alignment showing an example for the steps of the strategy diagram. Each sequence is computationally backtranslated to hypothetical nucleic acid sequences. IUPAC codes were used to show ambiguous positions. These sequences are piled up in order to get the degenerated consensus sequence. Numbers below this indicate the degeneration value of each position.

 The method presented here can be used starting with DNA or protein sequence alignments (Figure 1(a)). If the input was DNA, sequences were aligned to obtain one global degenerate DNA consensus. If the input was a protein alignment, each protein of the alignment is backtranslated into a degenerate DNA sequence. All the degenerate DNA sequences were combined in one global degenerate DNA consensus. This consensus sequence covers all the putative input sequences that could be the origin of each protein sequence (Figure 1(b)). Also, the consensus sequence may code for amino acids that were not detected in the known sequences. This is inevitable given the kind of degeneracy of the genetic code.

Then, the degenerate consensus sequence was analyzed using an overlapping window-based strategy. The window length corresponds to the required oligonucleotide length, and each window corresponds to a putative primer. For each candidate primer a score is calculated. In the first place, for each position of a candidate primer a position score (*Sp*_*i*_) was calculated using (1):

(1) $$\begin{array}{c} Sp_{i}=1-\text{lo}{\text{g}}_{10}\left(ND_{i}\right), \end{array}$$

where *ND*_*i*_ is the degeneracy value at the position *i* of the oligonucleotide (1 ≤ *i* ≤ *n*, where *n* is the length of the primer). *ND*_*i*_ is 1 for “*A*, *C*, *G* or *T*,” 2 for “*K*, *M*, *R*, *S*, *W* or *Y*,” 3 for “*B*, *D*, *H* or *V*,” and 4 for “*N*.” This expression takes a value of 1 for nondegenerate bases and decreases for more degenerated bases. On the other hand, it is known that in PCR reactions, the 3′ end of the primer is more important than the 5′ end. The region of the 3′ end of the primer must be as little degenerated as possible. Therefore, a good annealing at this end is imperative in order to minimize unspecific amplifications. Considering this, the value of *Sp*_*i*_ is multiplied by a weighting value (*Wp*_*i*_) defined by a straight line function that increases as it comes closer to the 3′ end (2):

(2) $$\begin{array}{c} Wp_{i}=pA+\frac{i\times \left(N_{y}-pA\right)}{N_{x}}, \end{array}$$

where *i* is the position from the 5′ end along the oligonucleotide (1 ≤ *i* ≤ *n*, where *n* is the length of the primer) and *pA*, *N*_*y*_, and *N*_*x*_ are user adjustable parameters defining the straight line function. *pA* is the axis intersection and (*N*_*y*_ − *pA*)/*N*_*x*_ is the slope. Default values for *pA*, *N*_*y*_, and *N*_*x*_ are 0, 1, and 1, respectively. Changing them will permit them to be more or less strict about including degenerations closer to the 3′ end of the primer. Increasing *pA* or *N*_*x*_, or decreasing *N*_*y*_, results in lesser stringency on the designed primer. Finally, to obtain a scaled global score (*S*_*g*_), the result of *Wp*_*i*_ × *Sp*_*i*_ is divided by the maximum possible score (*M*_*s*_, (3)). Global normalized score (*S*_*g*_) was calculated according to (4). In this way, *S*_*g*_ value varies from 0 to 1. Maximum score is obtained when the value of the *Sp*_*i*_ is 1 for each position. Therefore, *ND*_*i*_ must also be 1 too, and this only happened with nondegenerated primers:

(3) $$\begin{array}{c} M_{s}=n\times pA+\frac{\left(n+1\right)\times n\times \left(N_{y}-pA\right)}{2\times N_{x}}, \end{array}$$

(4) $$\begin{array}{c} S_{g}=\frac{\sum_{i=1}^{n}Sp_{i}\times Wp_{i}}{M_{s}}. \end{array}$$

## 3. Methods

### 3.1. Alignment and Sequence Comparison Tools

 For global alignment of protein sequences, the program ClustalW 1.83 [24] was used with default parameters. Local alignments of proteins against genomes were made using stand-alone Blast 2.2.13 [25] with default parameters. Oligonucleotide match searches were made with specifically developed tools written in C language. 

### 3.2. Sequence Data

 Several sets of sequences were used in the tests of the program, for designing and comparison of the primer sequences against genomes. All sequences GenBank's accession numbers are presented in Table 1.

### 3.3. Filtering Primers

 In addition to the scoring process, FAS-DPD can optionally filter the primers individually according to common criteria: melting point temperature (estimated using Santalucia's method [26]), *G* + *C* content, 5′ versus 3′ stability, presence of tandem repeats of the same base occurring at 3′ end or any place in the sequence, presence of a degenerated position at the 3′ end, and formation of homodimer structures. Also, primer pairs can be filtered according to amplification product size, melting point temperature compatibility, *G* + *C* content compatibility, and formation of heteroduplex structures. 

### 3.4. PCR Amplification

 The PCR conditions used in all experiments follow a common protocol. The reaction mix contained 1X Taq DNA polymerase buffer (Productos Bio-lógicos, Argentina), 0.2 mM dNTPs, 0.5 *μ*M of each primer, 20 pM template, and different concentration of MgCl_2_ and dimethyl sulfoxide (DMSO) in different reactions. The MgCl_2_ was used from 2 mM to 3 mM, and DMSO was used from 0% (v/v) to 5% (v/v). The reactions were performed in a total volume of 10 *μ*L, and the thermal profile consisted of an initial denaturation step of 94°C for 2 min, followed by 35 cycles of denaturation/annealing/extension steps. The denaturation step was at 92°C for 10 seconds, the temperature of the annealing step was not the same in all experiments, varying from 45°C to 60°C, and the time was always 15 seconds (see Figure 4). The extension step was at 72°C; the time of this step was 15 seconds. In all cases, one of the primers is specific for the template, while the other primer was designed by the method described in this work. The last step was a final extension of 5 minutes at 72°C. For Junin Virus, the template used was a plasmid containing a copy of cDNA of JUNV S genomic segment. For *Baculovirus*, the template was a plasmid containing a fragment of *Anticarsia gemmatalis MNPV* p74 gene. Sensitivity of the PCR assay was determined by dilution of cloned fragments from Junin virus [27] and *Baculovirus* template. 

## 4. Results

### 4.1. Distribution of Generated Primers

**Figure 2:**
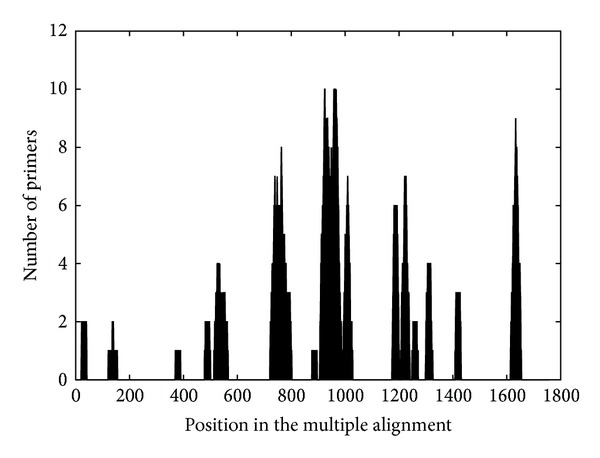
Primer distribution along one ORF. A collection of the best scoring primers for the nucleoprotein of *Arenavirus*, comprised of 50 primers for the genomic sequence and 50 for the antigenomic sequence, were represented in the corresponding alignment position. The height of each point indicates the cumulative number of primers corresponding at this position. The alignment was made with 71 arenavirus N protein sequences.

 The distribution of the resulting primers along the input sequence was analyzed. For this, the best one hundred primers obtained from a protein alignment were selected. For each position in the alignment, the number of the selected primers that correspond to this position was recorded (Figure 2). The test was repeated for different protein alignments.

The selected primers were located around a few hot spots in the alignment. This behavior indicates that there are generally few regions in a sequence alignment useful for degenerate primer design. Many primers found by the program are almost identical, shifting one or two bases between them, and located for most cases in a 30–40 base run. Similar results were obtained with all proteins tested. 

### 4.2. Intragenomic Specificity and Score Analysis

 Because it is possible that the best primers are not the less degenerated substrings in the collection of candidates, their specificity was tested. Also, it was necessary to get a more precise understanding of the score assigned by FAS-DPD in terms of specificity. To achieve this, the primers were compared with the complete genome sequences used to design them, looking for unspecific perfect matches.

For this task, a wide range of genome sizes was covered. Four collections of complete genome sequences were used: *Arenavirus* (genome in 10^4^ bases order), *Baculovirus* (genome in 10^5^ bases order), *Lactobacillus* (genome in 10^6^ bases order), and *Pseudomonas* (genome in 10^6^ bases order). For each set, a randomly selected genome was used as reference. Each annotated ORF of this genome was used to search related ORFs in the other genomes of the collection using the local Blast tool. The expected value of Blast was used to decide when two ORFs were related. When an ORF of the reference genome had a related one in all other genomes, all of them were aligned with ClustalW and used in further analysis.

Each resulting alignment was used as input for FAS-DPD to search primers. For each genome polarity the best fifty nonoverlapping primers were selected. This selection was made to avoid concentration of overrepresented, hot-spot-derived, high score primers. This allowed us to find a balanced set of primers, with high and low scores.

**Figure 3:**
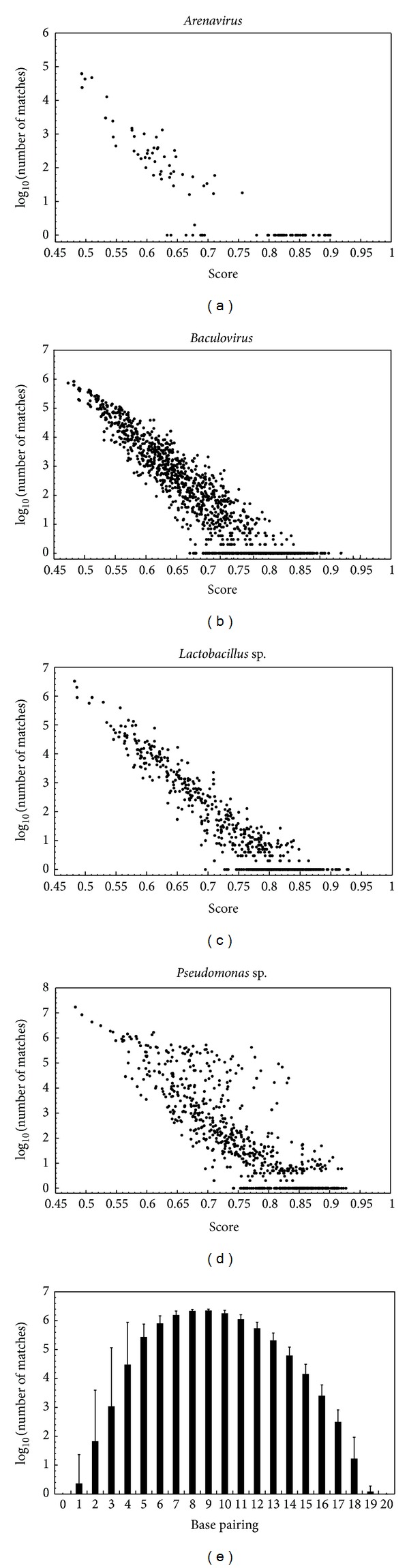
Specificity of primers. Primers designed for all ORFs shared among each model organism used were compared against the complete set of genomes for perfect matches with oligonucleotides of the same length. Each point represents the number of perfect matches (in log_10_ scale) of a primer in relation to its score. The length of the primers was 20 nucleotides. (a) *Arenavirus* genomes: 71 for S (small) RNA, 24 for L (large) RNA. (b) 22 *Baculovirus* genomes. (c) 5 *Lactobacillus *sp. genomes. (d) 7 *Pseudomonas *sp. genomes. (e) A set of primers for *Lactobacillus *sp. with scores between 0.85 and 0.90 were tested for nonperfect matches that could anneal unspecifically in PCR. Each bar represents the number of matches against the complete set of *Lactobacillus* genomes. The number below the bar indicates how many bases are shared.

In order to find the relationship between the score calculated for each primer and its specificity, all the primers were compared with all the oligonucleotides of the same size derived from each genome, searching for perfect matches (Figure 3). The results were similar for the four systems despite their differences in genome size.

There is an inverse correlation between primer score and the number of unspecific perfect matches. But this correlation is not linear. The quantity of unspecific perfect matches of primers with a minimal score of 0.85 and their target genome was generally zero. The number of unspecific perfect matches grew enormously with lower primer scores.

### 4.3. Experimental Challenge

 In addition to theoretic tests to determine the usefulness of FAS-DPD designed primers, experimental challenges were performed using *Arenavirus* and *Baculovirus* as models. The assay consisted in performing PCRs using a pair of primers, including a degenerated FAS-DPD designed primer and a standard nondegenerated primer (this allowed testing individually each designed primer), optimizing the reaction conditions and measuring its sensitivity.

**Figure 4:**
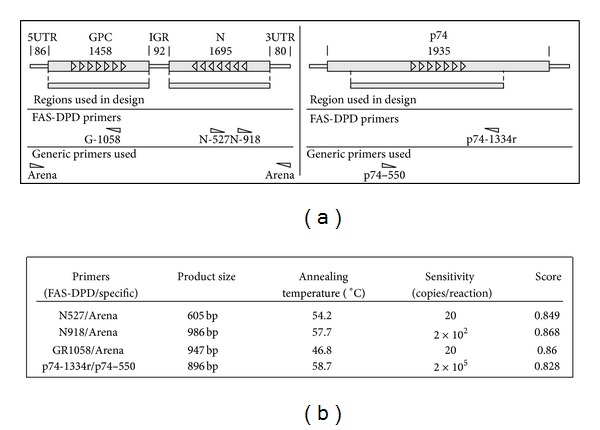
Experimental challenge of designed primers. (a) Genomic organization of the *Arenaviruses* S RNA and P74 ORF. *Arenavirus* shows an ambisense coding strategy of the GPC and N ORFs and three noncoding regions: 5′ untranslated region (5UTR), intergenic region (IGR), and 3′ untranslated region (3UTR). The location of each designed primer (GR1058, N918, N537, and p74-1334r) and specific primers (Arena, p74-550) is also shown. (b) The results obtained with each pair of primers tested and characteristics of reaction are shown.

For arenavirus, the primers were designed using sequences of 71 different GenBank records for the nucleoprotein (N protein) and the glycoprotein precursor (GPC protein). From the lists of the highest scored primers, three were randomly selected and synthesized for experimental evaluation, one for GPC (GR1058: RCNWHRTTNYCRAARCAYTT, score: 0.8596) and two for N (N527: GGNRYNSWNCCRAAYTGRTT, score: 0.8494; N918: NANRTTYTCRTANGGRTTNC, score: 0.8437) (Figure 4(a)).

 Amplification reactions were performed using each of these primers together with the Arena primer CGCACCGGGGATCCTAGGC) as nondegenerated counterpart. The latter is a generic primer for *Arenaviruses* that matches almost perfectly with the nineteen bases of 3′ end of the genomic RNA sequence and with the nineteen bases of 3′ end of the antigenomic RNA sequence of all known arenaviruses. The reaction template was a cDNA corresponding to the Junin virus small RNA segment which encodes the N and GPC proteins.

For *Baculovirus*, one primer (p74-1334r: BYRWRNCCVWRNGGRTCSCA, score: 0.8281) was designed using 57 sequences of p74 different *Baculovirus*. As its counterpart, a specific primer for *Anticarsia gemmatalis MNPV* was used [28] (p75-550r: GGcGTGGACGACGTGC). The reaction template was the *Anticarsia gemmatalis MNPV* p74 isolate 2D [29] gene cloned in a plasmid.

PCRs were assayed with different sets of conditions, and the sensitivity was measured. Sensitivity achieved with arenavirus primers was high. Twenty copies/*μ*L or less of specific template were detected. For *Baculovirus* the detection was not as sensible as for arenavirus, but it can be considered as a good sensitivity; 2 × 10^4^ copies/*μ*L of specific template were detected. This difference can be explained taking into account that the divergence observed for baculovirus sequences is greater than for arenavirus. Therefore, the score for the p74-1334r primer was lower than that of *Arenavirus*.

### 4.4. Increment of Degeneration of FAS-DPD Designed Primers in relation to Minimum Degenerated Substring

**Figure 5:**
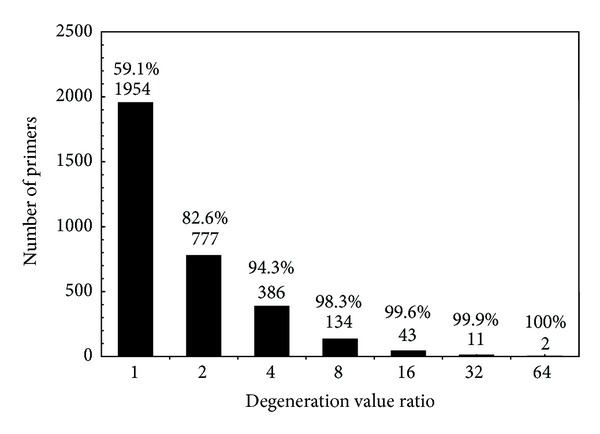
Comparison of FAS-DPD designed primers and minimum degenerated substrings. Collection of primers with the highest score designed for all the ORFs shared by all the genomes used were compared against the minimum degenerated subsequence of the same length for each ORF in order to know how much more degenerated they are. The number below each bar indicates the ratio of degeneration between the designed primer and the minimum degeneration substring. The number above each bar indicates the amount of primers that correspond with the ratio mentioned before. The percentages are cumulative with respect to increasing degeneration ratios and referred to the total number of primers used in the test.

 The aim of FAS-DPD is to design universal degenerated primers that are not necessarily the less degenerated sequences of the collection of candidates. In order to know how much degeneration FAS-DPD designed primers acquire, another test was performed. Given an alignment of homologous ORFs, the degeneration was calculated for the highest scoring primer selected with FAS-DPD and for the minimum degenerated substring of the same length. Then, the ratio of these two values was obtained. The comparison was made with the complete set of ORF alignments used before (*Arenavirus*, *Baculovirus*, *Pseudomonas,* and *Lactobacillus*) (Figure 5). In more than 90% of the cases the increase of degeneration value is at most fourfold (e.g., changing “…A…” to “…N…” or “…A … A…” to “…R … W…”). Therefore, these primers have only up to two more degenerated positions than the substring with minimum degeneration.

It is important to note that, in general, there is not only one minimum degeneracy substring for each ORF. The decision of which primer is better must not only take into account the degeneration value. The position of degenerated bases in the sequence is crucial. The ratio of greater increase of degeneration found was 64; this corresponds to only less than 0.1% of primers. This result shows that FAS-DPD primers are more degenerated than the less degenerated substring, but this increase of degeneration is slight and does not imply a high compromise of the specificity. 

## 5. Discussion

 In this work we presented a new algorithm, implemented in the FAS-DPD software, as an alternative strategy to solving DPD problems. FAS-DPD was designed to use multiple alignments of proteins or nucleic acids as input data and constructs a consensus degenerate sequence from that, which is then used to design the putative primers.

The experimental background knowledge from molecular biology teaches us that in the real world the 3′ ends of primers are key determinants of a successful amplification. FAS-DPD takes into account this property and incorporates special considerations in the global score calculation becoming more strict for the 3′ end than for the 5′ end.

The specificity of the set of primers designed with FAS-DPD was computationally tested with several collections of whole genomes, ranging from 10^4^ bp to 10^6^ bp. The restriction to higher lengths was due to the lack of whole genome collections for genus of bigger sizes with several individuals. In all genome collections assayed the results showed the same behavior; there is a relationship between the score value and the number of unspecific perfect matches. This analysis allows us to suggest a cut-off score (0.85) for primers that could be more successful.

PCRs were successfully performed on arenaviral and baculoviral models. For arenavirus, the designed GPC or N primers were used with the universal Arena primer [30]. For *Baculovirus*, the designed p74 primer was used with a specific p74 primer [28]. Each reaction was tested in different conditions in order to optimize its yield.

FAS-DPD software is licensed under GNU General Public License Version 3 and is available at http://www.github.com/javieriserte/fas-dpd.

In general, the results suggest that FAS-DPD could be used to design generalized degenerate primers for detection of known or unknown members of gene families or organism families, including different types of pathogens. Also, this tool would allow a more efficient search for enzymes and other proteins with commercial or biotechnological importance, making for a faster and cheaper research process.

## Figures and Tables

**Table 1:** List of sequences used in the test of FAS-DPD. Accession numbers and brief description are presented.

Acc. number	Sequence description	Acc. number	Sequence description
Arenaviral sequences			

AY129248.1	Machupo v. st. Carvallo	U41071.1	Sabia v.
AF485260.1	Machupo v. st. Carvallo	EU260463.1	Chapare v. st. 810419
AY924206.1	Machupo v. st. MARU-216606	AY081210.1	Allpahuayo v. CLHP-2098
AY924202.1	Machupo v. st. Chicava	AY012686.1	Allpahuayo v. from Peru
AY624355.1	Machupo v. st. Chicava	AY012687.1	Allpahuayo v. st. CLHP-2472
AY924205.1	Machupo v. st. 9301012	AF485262.1	Pirital v. st. VAV-488
AY619645.1	Machupo v. st. Mallele	AF277659.1	Pirital v.
AY924203.1	Machupo v. st. 9430084	M16735.1	Pichinde v.
AY924208.1	Machupo v. st. MARU 249121	AF485261.1	Parana v. st. 12056
AY924204.1	Machupo v. st. 200002427	AF512829.1	Parana v. st. 10256
AY924207.1	Machupo v. st. MARU 222688	AF512831.1	Flexal v. st. BeAn 293022
AY571959.1	Machupo v. st. 9530537	AF485257.1	Flexal v. st. Pinheiro
AY746353.1	Junin v. st. Candid-1	AF512831.1	Flexal v. st. BeAn 293022
AY358023.2	Junin v. st. XJ13	AF512830.1	Latino v. st. MARU 10924
AY619641.1	Junin v. st. Rumero	AF485259.1	Latino v. st. Maru 10924
D10072.2	Junin v. st. MC2	U34248.1	Oliveros v.
M20304.1	Tacaribe v.	AY847350.1	LCM v. st. Armstrong 53b
AF485256.1	Amapari v. st. BeAn 70563	M20869.1	LCM v. st. Armstrong 53b
AF512834.1	Amapari v. st. BeAn 70563	EU136038.1	Dandenong v. is. 0710-2678
AF512832.1	Cupixi v. st. BeAn 119303	DQ328874.1	Mopeia v. st. Mozambique
AY129247.1	Guanarito v. st. INH-95551	DQ328877.1	Ippy v. st. Dak-An-B-188-d
AF485258.1	Guanarito v. st. INH-95551	X52400.1	Nigeria Lassa v.
AY497548.1	Guanarito v. st. CVH-960101	AY628206.1	Lassa v. st. Weller
AY924392.1	Bear Canyon v. st. AV 98470029	AY628201.1	Lassa v. st. Macenta
AY924391.1	Bear Canyon v. st. AV A0070039	AY628205.1	Lassa v. st. Z148
AF512833.1	Bear canyon v. st. A0060209	J04324.1	Lassa v. st. Josiah
DQ865244.1	Catarina v. st. AV A0400135	AY772168.1	Mopeia Lassa reassortant 29
DQ865245.1	Catarina v. st. AV A0400212	AY628203.1	Lassa v. st. Josiah
EU123328.1	Skinner Tank v. st. AV D1000090	AF181853.1	Lassa v. st. LP
EU123331.1	North American arenav. st. AV 96010024	AY628207.1	Lassa v. st. Pinneo
EU123330.1	North American arenav. st. AV 96010151	AY628208.1	Lassa v. st. Acar-3080
AF228063.1	Whitewater Arroyo v. st. 9310135,	AF181854.1	Lassa v. st. 803213
AF485264.1	Whitewater Arroyo v. st. 9310141	AY342390.1	Mobala v. st. ACAR-3080-MRC5-P2
EU123329.1	North American arenav. st. AV D1240007	M33879.1	Mopeia v. st. AN-21366
AF485263.1	Tamiami v. st. CDC W-10777	AY772170.1	Mopeia v. st. AN-20410
AF512828.1	Tamiami v. st. W 10777		

Baculoviral sequences			

AP006270.1	*Adoxophyes honmai* nucleopolyhedrovirus DNA	X77048.1	*Cryptophlebia leucotreta* granulosis
AF547984.1	*Adoxophyes orana* granulovirus	X79569.1	*Cryptophlebia leucotreta* granulosis
NC_005839.2	*Agrotis segetum* granulovirus	NC_002816.1	*Cydia pomonella* granulovirus
L22858.1	*Autographa californica* nucleopolyhedrovirus clone C6	NC_003083.1	*Epiphyas postvittana* NPV
L33180.1	*Bombyx mori* nuclear polyhedrosis virus isolate T3	NC_002654.2	*Helicoverpa armigera *
NC_005137.2	*Choristoneura fumiferana* DEF MNPV	AF081810.1	*Lymantria dispar *
NC_004778.3	*Choristoneura fumiferana* MNPV	NC_003529.1	*Mamestra configurata* NPV-A
AY864330.1	*Chrysodeixis chalcites* NPV	U75930.2	*Orgyia pseudotsugata* MNPV
AY456389.1	*Chrysodeixis chalcites* NPV	AF499596.1	*Phthorimaea operculella* granulovirus
AY456390.1	*Chrysodeixis chalcites* NPV	NC_002593.1	*Plutella xylostella* granulovirus
AY545786.1	*Chrysodeixis chalcites* NPV	NC_004323.1	*Rachiplusia ou* MNPV
AY545787.1	*Chrysodeixis chalcites* NPV	NC_002169.1	*Spodoptera exigua* MNPV
AY229987.1	*Cryptophlebia leucotreta* granulovirus	NC_003102.1	*Spodoptera litura* NPV
AY096241.1	*Cryptophlebia leucotreta* granulovirus	NC_007383.1	*Trichoplusia ni* SNPV
AY096242.1	*Cryptophlebia leucotreta* granulovirus		

*Pseudomonas *sp. sequences			

NC_007492.2	*Pseudomonas fluorescens* Pf0-1	NC_004578.1	*Pseudomonas syringae *
NC_005773.3	*Pseudomonas syringae *	NC_002947.3	*Pseudomonas putida *
NC_004129.6	*Pseudomonas fluorescens *	NC_002516.2	*Pseudomonas aeruginosa *
NC_007005.1	*Pseudomonas syringae *		

*Lactobacillus *sp. sequences			

NC_005362.1	*Lactobacillus johnsonii *	NC_002662.1	*Lactococcus lactis* subsp.
NC_007576.1	*Lactobacillus sakei* subsp.	NC_004567.1	*Lactobacillus plantarum *
